# Oral supplementation with *Lactobacillus fermentum* MC018 improves intestinal health, immune response, and growth performance of Zi geese infected with *Escherichia coli* XH197291

**DOI:** 10.3389/fvets.2025.1666985

**Published:** 2025-09-02

**Authors:** Yang Li, Meng Liu, Zehao Li, Meiqi Dong, Linru He, Peilong Li, Ruosi Chen, Yue Liang, Lijia Yang, Fei Li, Yulong Zhou, Zhanbo Zhu, Yu Liu

**Affiliations:** College of Animal Science and Veterinary Medicine, HeiLongJiang BaYi Agricultural University, Daqing, China

**Keywords:** geese, *Lactobacillus fermentum*, *Escherichia coli*, intestinal health, growth performance

## Abstract

**Introduction:**

*Escherichia coli* infection causes severe diarrhea, decreases growth performance, and increases mortality of poultry, which imposes a significant economic burden on the poultry industry and severely limits its growth.

**Methods:**

Here, to investigate the effects of *Lactobacillus* on the intestinal health, immune response, and growth performance of *E. coli*-infected goslings, we established a geese model infected with an *Stx2f* gene-carrying *E. coli* strain and analyzed the probiotic characteristics of three *Lactobacillus* isolates obtained from the cecum of healthy geese. In an *in vivo* study, Zi geese were administered daily gavage of *L. johnsonii* MC006, *L. salivarius* MC013, or *L. fermentum* MC018 (10^9^ CFU/mL) from 1 d of age for 21 d, followed by treatment with *E. coli* XH197291 gavage (10^9^ CFU/mL) on day 8.

**Results:**

The results showed that *E. coli* XH197291-infected geese exhibited depression, intestinal damage, reduced average daily gain, increased feed conversion ratio, and 100% diarrhea incidence within 48 h post-infection. Remarkably, among the three *Lactobacillus* isolates, *L. fermentum* MC018 showed the potential to function as a probiotic because of its ability to resist acid and bile degradation, antibacterial effect, and adhesion property. Notably, oral supplementation containing *L. fermentum* MC018 alleviated diarrhea and intestinal histological lesions, reduced *E. coli* counts in both ileum and rectum, increased the population of lactic acid bacteria, and improved the growth performance of *E. coli*-infected geese. Geese treated with *L. fermentum* MC018 gavage had higher serum diamine oxidase (*p* < 0.01) and IgM (*p* < 0.05) levels than those in the model group. *L. fermentum* MC018 reduced the levels of IL-1β, IL-6, and TNF-*α* in intestinal tissues following *E. coli* infection. Compared to *L. salivarius* MC013, *L. fermentum* MC018 increased the levels of ZO-1 in the duodenum and Claudin-1 in the ileum.

**Discussion:**

These findings suggest that *L. fermentum* MC018 is a promising probiotic strain for use as a potential alternative to antibiotics for controlling avian colibacillosis.

## 1 Introduction

Avian pathogenic *Escherichia coli* (APEC) infection can cause avian colibacillosis, leading to severe diarrhea, decreased feed intake, reduced growth performance and egg production, and increased mortality of poultry (1). Avian colibacillosis is a key infectious disease in poultry farms with a broad global distribution and has resulted in major economic losses to the poultry industry (2). Infected poultry and contaminated poultry meat or eggs as pathogen carriers pose a serious threat to human health and food safety (3). Because of the intense pressure on the livestock and poultry feed industry to limit or ban antibiotic usage (4, 5), it is very critical to explore new strategies and alternatives for antibiotics to prevent and control APEC infection in poultry for improving their intestinal health and growth performance (6).

*Lactobacillus*, as a live microbial feed supplement, could serve as a potential alternative strategy for antibiotic use in poultry feed (7, 8). *Lactobacillus* can also improve intestinal barrier function (9), modulate immune response (10, 11), and enhance growth performance (12). Several *Lactobacillus* species, for example, *L. acidophilus*, *L. plantarum*, *L. fermentum*, *L. salivarius*, and *L. johnsonii*, are known for their remarkable probiotic qualities (13–16). Previous studies have shown that *L. acidophilus* CGMCC14437 and *L. plantarum* B1 improved intestinal barrier function and growth performance and suppressed *Escherichia coli* infection in broiler chickens (3, 17). However, the role of *Lactobacillus* in attenuating *E. coli* infection in geese remains unclear.

Hence, in the present study, we established a geese model of *E. coli* infection by referring to previous studies (3, 17). Next, we isolated and identified three *Lactobacillus* strains from the cecal content of healthy Zi geese, evaluated their biological characteristics, and investigated the effects of oral supplementation of these *Lactobacillus* strains on the intestinal health, immune response, and growth performance of the *E. coli*-infected geese model. The findings suggest that *L. fermentum* MC018 could serve as a promising alternative for antibiotics in controlling avian colibacillosis.

## 2 Materials and methods

### 2.1 Ethics statement

The study protocol was approved by the Institutional Animal Care and Use Committee of HeiLongJiang BaYi Agricultural University (Approval No. DWKJXY2024231). The study was conducted in accordance with the local legislation and institutional requirements.

### 2.2 Isolation and identification of *Lactobacillus* strains from geese cecal content

By using labeled sterile tubes, cecal content samples were collected from three healthy Zi geese slaughtered at the age of 3 months in a local farm of Daqing in Heilongjiang Province, China. The Zi goose, an excellent local breed in Northeast China, boasts advantages including cold resistance, tolerance for rough forage, high-quality meat, and high egg production (18). The samples were serially diluted in physiological saline (0.85% NaCl, w/v) and cultured on De Man-Rogosa-Sharpe (MRS, Aobox, China) agar plates under anaerobic conditions at 37 °C for 24–48 h for bacterial isolation. The isolated colonies were randomly selected based on their morphology and analyzed by Gram staining and optical microscopy. The isolated strains were identified by PCR and phylogenetic analysis. The following universal primers were used for amplifying 16S rRNA sequences of the strains based on a previous study (19): forward (5′-AGA GTT TGA TCC TGG CTC AG − 3′) and reverse (5′-GGT TAC CTT GTT ACG ACT T-3′).

PCR was performed in a 25-μL reaction mixture containing 1 μL of DNA, 12.5 μL of 2 × Taq Master Mix (Qiagen, Germany), 0.5 μL of each primer (10 μM), and 10.5 μL of nuclease-free water. For each PCR reaction, a negative control containing sterile double distilled water was used. The mixture was amplified under the following conditions: initial denaturation at 95 °C for 7 min, followed by 35 cycles of denaturation at 94 °C for 60 s, annealing at 56 °C for 60 s, and elongation at 72 °C for 90 s and the final elongation step at 72 °C for 10 min. The amplified PCR products were analyzed by 1.5% agarose gel electrophoresis, and positive bands were detected using an ultraviolet trans-illuminator. All PCR products were sent to Harbin Ruibiotech Biotechnology Co., Ltd. for DNA sequencing. The 16S rRNA sequences of the isolated strains were subjected to NCBI BLAST search, and the phylogenetic tree was constructed using MEGA7.0 software.

### 2.3 Characterization of the isolated *Lactobacillus* strains

Growth and pH curves were used to evaluate the growth and acid production ability of the isolated *Lactobacillus* strains, respectively. A single colony was selected and inoculated into 5 mL MRS broth, and the broth was incubated at 37 °C for 24 h to obtain purified cultures. The purified strains were inoculated into MRS broth at 1:100 dilution for 36 h, and the OD_600_ value and pH of the culture medium were measured every 2 h. The growth and pH curves were plotted based on the measured data.

The antibacterial ability of the isolated strains was measured using their suspension and culture supernatant by the Oxford cup method. The purified cultures were transferred to a 5 mL MRS broth with 1% inoculation and grown for 24 h. The suspension was centrifuged at 10,000 rpm for 5 min. The supernatant was collected and used for the subsequent bactericidal test. Four goose-derived pathogenic *E. coli* strains (XH197291, GenBank: PV426822.1; QE191291, GenBank: PV535338.1; BA220820, GenBank: PV535342.1; BA220826, GenBank: PV535340.1) preserved in our laboratory were used to determine the antibacterial activity of the isolated *Lactobacillus* strains. These *E. coli* strains were evenly spread on nutrient agar plates at the cell density of 1 × 10^6^ CFU/plate. Approximately 200 μL of the supernatant was added to each Oxford cup (Tuopu, China), and the plates were incubated at 37 °C for 12 h. The size of the inhibition zone was then measured.

The ability of the isolated strains to resist degradation by acid and bile salt was evaluated by a survival assay with MRS broth at pH 2.0 and 3.0 or containing bile salt at concentrations of 0.2 and 0.3%, as reported previously (20). Cells of the purified strains were collected by centrifugation, washed twice with phosphate-buffered saline (PBS), and resuspended in MRS broth with pH 2.0 and 3.0 or containing bile salt at concentrations of 0.2 and 0.3%. The cell suspensions (2 × 10^8^ CFU/mL) were incubated at 37 °C for 3 h. The survival rate was defined as the ratio of the OD_600_ value after treatment to the initial OD_600_ value. Data are presented as mean ± SD of triplicates from 3 independent experiments.

The surface hydrophobicity (H%) and auto-aggregation (Auto-A%) abilities were tested based on previous studies (21, 22). Cells of the isolated strains were collected by centrifugation, washed, and resuspended in sterile PBS to achieve an absorbance reading of 0.8 (OD_initial_) at 600 nm. Next, 3 mL of the suspension was mixed with 1 mL of xylene and incubated at 37 °C for 10 min. The mixture was vortexed briefly and incubated at 37 °C for 3 h for phase separation. The aqueous phase was gently removed for measuring absorbance (OD_final_) at 600 nm. Surface hydrophobicity (H%) was calculated using the following equation: H% = (OD_initial_ − OD_final_)/OD_initial_ × 100%. Additionally, bacterial cells were collected again and resuspended in PBS to achieve an absorbance value of 0.8 (OD_initial_) at 600 nm. The suspension was incubated at 37 °C for 3 h. The upper suspension was used to measure the absorbance reading (OD_final_) at 600 nm. Auto-A% was calculated using the following equation: Auto-A% = (OD_initial_ − OD_final_)/OD_initial_ × 100%.

To conduct the hemolysis test, the purified strains were streaked on agar plates containing 5% sheep blood. The plates were then incubated at 37 °C for 24 h and examined for the presence of green-hued zones (*α*-hemolysis), white or transparent zones (β-hemolysis), or the absence of hemolysis (*γ*-hemolysis) around the colonies. *Staphylococcus aureus* DQ13160 (GenBank: PV535337.1) was used as a positive control. The *in vivo* safety was also evaluated using one-day-old healthy Zi geese. Each goose in all *Lactobacillus*-treated groups was orally gavaged with 1 mL of the corresponding purified bacterial suspension at 10^9^ CFU/mL cell density for 7 continuous days (d). At the end of the experiment, all geese were euthanized by intravenous sodium pentobarbital (100 mg/kg of body weight) injection as reported previously (3). Clinical signs and anatomical changes in the geese were examined and compared with the control group (PBS-fed geese).

### 2.4 Animals and experimental design

First, a goose model of *E. coli* infection was established by referring to previous reports (3, 23). A total of 648 one-day-old Zi geese were adaptively reared until 8 d of age. The geese were then randomly assigned to nine groups: *E. coli* XH197291-infected group (1 mL, 1 × 10^8^ CFU/mL), *E. coli* XH197291-infected group (1 mL, 1 × 10^9^ CFU/mL), *E. coli* QE191291-infected group (1 mL, 1 × 10^8^ CFU/mL), *E. coli* QE191291-infected group (1 mL, 1 × 10^9^ CFU/mL), *E. coli* BA220820-infected group (1 mL, 1 × 10^8^ CFU/mL), *E. coli* BA220820-infected group (1 mL, 1 × 10^9^ CFU/mL), *E. coli* BA220826-infected group (1 mL, 1 × 10^8^ CFU/mL), *E. coli* BA220826-infected group (1 mL, 1 × 10^9^ CFU/mL), and mock-infected group. Six replicates per group and 12 geese per replicate were used. During the experiment, all geese were kept in cages and fed a commercial antibiotic-free, corn-soybean meal-based gosling diet (nutrient levels of the diet are shown in Supplementary Table S1) and had free access to feed and drinking water every day. The indoor temperature was maintained at 30–31 °C in the first week and then decreased by 2–3 °C per week, and the ambient humidity was controlled at 60–65% (24, 25). Each goose in the eight infected groups was orally gavaged with 1 mL of the corresponding *E. coli* suspension at 10^8^ or 10^9^ CFU/mL cell density, and the geese in the mock-infected group were administered 1 mL of PBS. Clinical signs and anatomical changes were observed within 7 d after *E. coli* infection. Blood samples were collected from the heart and centrifuged to obtain the serum. All experimental geese were then euthanized by intravenous sodium pentobarbital (100 mg/kg of body weight) injection to collect intestinal tissue and anal swab samples as reported previously (3). The effects of *E. coli* infection were evaluated through clinical, bacteriological, and histopathological assessments. The diarrhea rate was reported as the percentage of geese with diarrhea.

Subsequently, based on the *E. coli* XH197291 infection model, we evaluated the effects of three goose-derived *Lactobacillus* strains on the growth performance, intestinal health, and immune response of Zi geese. A total of 360 one-day-old Zi geese (initial body weight: 101.6 ± 2.3 g) were randomly assigned to five groups: *E. coli* XH197291-infected model group, *E. coli* XH197291 + *L. johnsonii* MC006 group, *E. coli* XH197291 + *L. salivarius* MC013 group, *E. coli* XH197291 + *L. fermentum* MC018 group, and mock-infected group. Six replicates per group and 12 geese per replicate were used. During 1–7 d of age, each goose in the mock-infected and model groups was administered 1 mL of PBS, and the geese in the three *Lactobacillus*-supplemented groups were orally gavaged with 1 mL of the corresponding *Lactobacillus* suspension at 10^9^ CFU/mL cell density (7). At 8 d of age, each goose in the model group and three *Lactobacillus*-supplemented groups was orally gavaged with 1 mL of *E. coli* XH197291 suspension at 10^9^ CFU/mL cell density. During 8–21 d of age, each goose in the three *Lactobacillus*-supplemented groups was orally gavaged with 1 mL of the corresponding *Lactobacillus* suspension at 10^9^ CFU/mL cell density. Clinical signs, diarrhea rate, and growth performance were recorded and analyzed during the experiment. Intestinal tissues, anal swab samples, and blood samples were collected for subsequent analyses.

### 2.5 Histological analysis

Segments of the duodenum and ileum were collected at 48 h post-infection (hpi) and fixed with 4% paraformaldehyde. Next, 4- to 6-mm-thick paraffin-embedded tissue sections were stained with hematoxylin and eosin. The sections were then examined under a light microscope. Five different villi and crypts per section were measured to assess villus height and crypt depth, and the ratio of villus height to crypt depth **(V/C)** was determined.

### 2.6 PCR detection for *Stx2f* gene

To detect the presence of *Stx2f* gene-carrying *E. coli* XH197291 used for challenge in the model group, anal swab samples from each goose in the mock-infected and model groups were tested by PCR for the *Stx2f* gene at 48 hpi. The following self-designed specific primers were used for amplifying the *Stx2f* gene (GenBank: PX099224): forward (5′-ATG ACG ACG GAC AGC AGT T-3′) and reverse (5′-CAA AGT GCT CAG CTG ACA GGG-3′). PCR cycling conditions included an initial denaturation step at 95 °C for 5 min, followed by 34 cycles at 94 °C for 60 s, 58 °C for 60 s, and 72 °C for 60 s and the final elongation step at 72 °C for 6 min. The amplified PCR products were visualized by 1.5% agarose gel electrophoresis.

### 2.7 *E. coli* and lactic acid bacteria (LAB) count

In accordance with previous studies (17, 26), 0.5 g of ileal or rectal contents were diluted with 4.5 mL of sterile PBS and then serially diluted 10-fold from 10^−1^ to 10^−8^. The contents were plated on MacConkey’s agar (02-005 K, Aobox, Beijing, China) to culture *E. coli*, and the agar plates were incubated at 37 °C for 24 h; the contents were also plated on MRS agar to culture LAB, and the agar plates were incubated under anaerobic conditions at 37 °C for 24 h. All bacteria were enumerated (CFU/g contents) by a visual count of colonies. The colony counts were log transformed before statistical analysis.

### 2.8 Quantitative real-time PCR (qRT-PCR) analysis

Total RNA was extracted from the duodenum and ileum tissue samples by using TRIzol reagent (15596026CN, Invitrogen Life Technologies, Carlsbad, CA) in accordance with the manufacturer’s instructions. The mRNA expression levels of Claudin-1, Occludin, ZO-1, IL-1β, IL-6, and TNF-*α* in the duodenum and ileum at 48 hpi were measured by qRT-PCR with the CFX96 Touch Real-Time PCR Detection System (Bio-Rad, Hercules, CA) using SYBR Premix Ex Taq II (RR820A, TaKaRa Biotechnology, Dalian, China). GAPDH was used as the internal control gene, with self-designed specific primers (Supplementary Table S2). The reaction cycling conditions were as follows: initial denaturation at 95 °C for 30 s, followed by 40 cycles of 95 °C for 5 s, 60 °C for 30 s, and 70 °C for 30 s. A final melting curve analysis was performed from 65 °C to 95 °C at the rate of 0.1 °C/s (continuous acquisition). Each sample was tested in triplicate, and the fold differences in gene expression were calculated using the 2^−ΔΔCt^ method with normalization to GAPDH.

### 2.9 Western blot analysis

Protein levels of Claudin-1, Occludin, and ZO-1 in the duodenum and ileum at 48 hpi were measured by Western blotting. Total protein was extracted from the duodenum and ileum tissue samples with 100–150 μL of radioimmunoprecipitation assay buffer (Beyotime, Shanghai, China) containing 15 mM phenylmethylsulfonyl fluoride (Beyotime, Shanghai, China), before quantifying using a bicinchoninic acid assay kit (P0012S, Beyotime, Shanghai, China) in accordance with the manufacturer’s instructions. Approximately 30 μg of total protein was separated by electrophoresis and transferred to a polyvinylidene fluoride membrane (0.45 μm; EMD Millipore, Billerica, MA, USA), which was blocked with 5% non-fat milk in TBST (Tris-buffered saline, NaCl, and Tween 20) for 1–2 h at room temperature, and then incubated with primary antibodies including anti-Claudin-1 (GB112543, Servicebio, Wuhan, China), anti-Occludin (GB11149, Servicebio, Wuhan, China), anti-ZO-1 (GB111402, Servicebio, Wuhan, China), and anti-GAPDH (10494-1-AP, Proteintech, Rosemont, USA) overnight at 4 °C. After three rinses for 15 min with TBST, the membranes were incubated with horseradish peroxidase (HRP)-conjugated AffiniPure goat anti-rabbit IgG (H + L) (SA00001-2, Proteintech) for 1 h at room temperature. Subsequently, the membranes were rinsed three times for 15 min with TBST. Finally, the protein bands were visualized using a chemiluminescent HRP substrate (EMD Millipore), quantified using a Western blot imaging system (e-Blot Touch Imager; e-BLOT Life Science, Shanghai, China), and compared using GraphPad Prism version 8.0 (GraphPad Software, Inc.).

### 2.10 ELISA analysis

At 48 hpi, 400 μL of blood sample was collected from the heart and centrifuged to obtain the serum. The serum levels of IgA (kit No. YX-090701G), IgM (kit No. YX-090714G), and DAO (kit No. YX-040115G) were determined in accordance with the manufacturer’s instructions (Sinobestbio, Shanghai, China). Meanwhile, the production of IL-1β (kit No. ml061217), IL-6 (kit No. ml061135), and TNF-*α* (kit No. ml036890) in the duodenum and ileum was measured following the manufacturer’s instructions (MLbio, Shanghai, China).

### 2.11 Growth performance analysis

The body weight and feed intake of geese were measured before morning feeding at 1, 7, 8, and 21 d of age. Average daily gain (ADG), average daily feed intake (ADFI), and feed conversion ratio (feed: body weight gain, g: g, FCR) were calculated at different experimental periods (1–7 d and 8–21 d).

### 2.12 Statistical analysis

All data are expressed as mean ± standard deviation and analyzed with Student’s unpaired t-test, one-way ANOVA, or two-way ANOVA by using GraphPad Prism version 8.0 (GraphPad Software, Inc.). A *p*-value of <0.05 was considered statistically significant. All samples were assayed in triplicate.

## 3 Results

### 3.1 Identification of *Lactobacillus* strains

**Figure 1:**
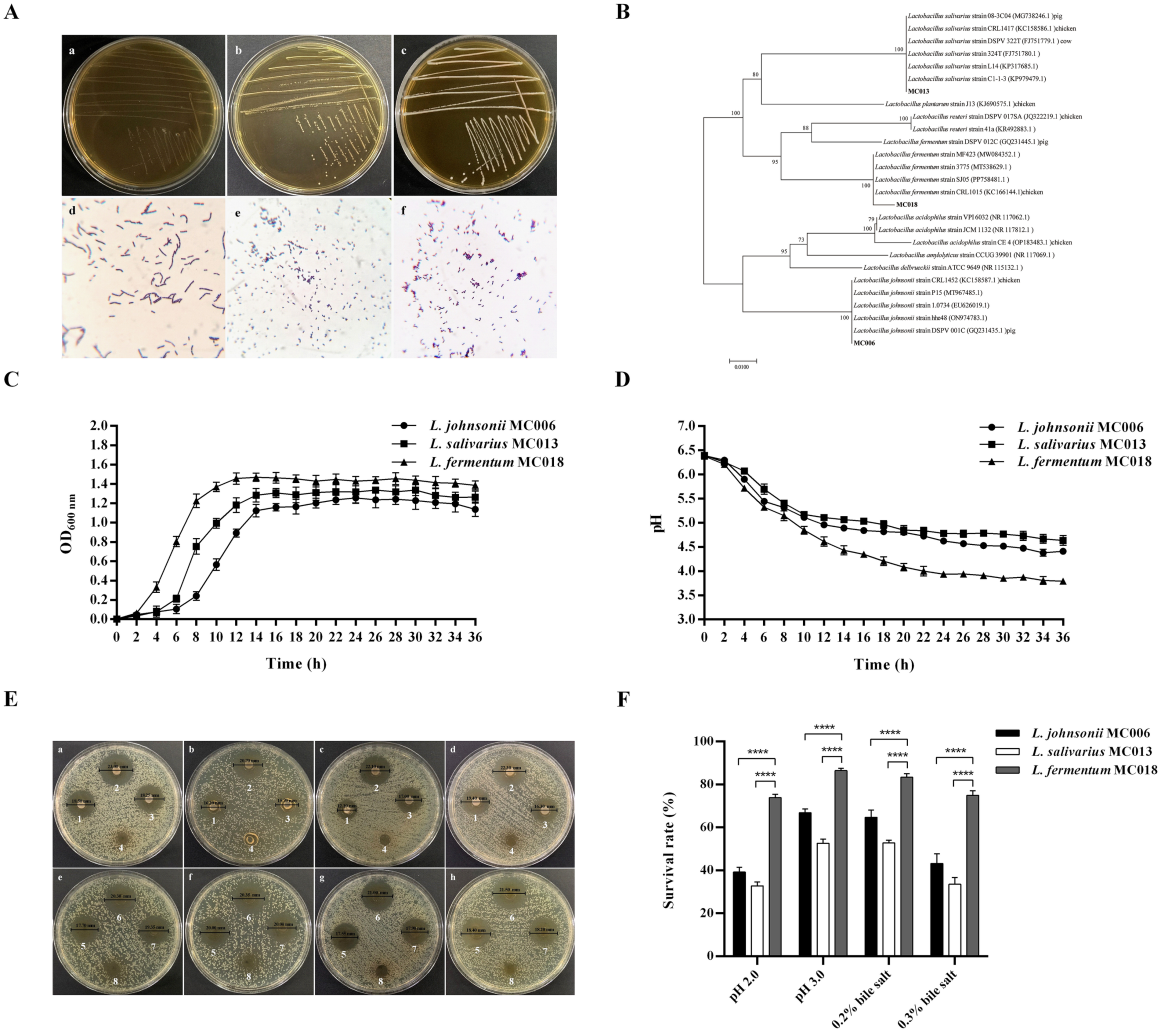
Identification and characterization of three goose-derived *Lactobacillus* strains. **(A)** Colony characteristics and Gram staining. Colony characteristics: (a) *L. johnsonii* MC006 colonies, (b) *L. salivarius* MC013 colonies, (c) *L. fermentum* MC018 colonies. Gram staining (original magnification, 1,000×): (d) *L. johnsonii* MC006, (e) *L. salivarius* MC013, (f) *L. fermentum* MC018; **(B)** Phylogenetic tree; **(C)** Growth curves; **(D)** pH curves; **(E)** Antibacterial ability of the isolated strains against *E. coli* was evaluated by the Oxford cup method. Bacterial suspensions of *L. johnsonii* MC006 (1), *L. fermentum* MC018 (2), or *L. salivarius* MC013 (3) were used for inhibiting *E. coli* XH197291 (a), *E. coli* QE191291 (b), *E. coli* BA220820 (c), or *E. coli* BA220826 (d). The supernatants of *L. johnsonii* MC006 (5), *L. fermentum* MC018 (6), or *L. salivarius* MC013 (7) were used for inhibiting *E. coli*, XH197291 (e), *E. coli* QE191291 (f), *E. coli* BA220820 (g), or *E. coli* BA220826 (h). MRS broth was used as the control (4, 8); **(F)** Acid and bile salt resistance characteristics, ****p < 0.0001. Data are presented as mean ± SD (*n* = 3 per group) and analyzed using two-way ANOVA.

We isolated 3 strains of Gram-positive bacilli, designated as strain MC006 (GenBank: PV426770.1), strain MC013 (GenBank: PV426771.1), and strain MC018 (GenBank: PV426772.1). The strains exhibited good growth in MRS medium (Figures 1A,C). The colony morphology of these strains on MRS agar and their Gram staining characteristics are shown in Figure 1A. Phylogenetic analysis (Figure 1B) revealed that strains MC006, MC013, and MC018 belonged to *L. johnsonii*, *L. salivarius*, and *L. fermentum*, respectively.

### 3.2 Characterization of the *Lactobacillus* strains

As shown in Figure 1C, the three *Lactobacillus* strains were in the logarithmic growth phase from 4 to 14 h, stable from 14 to 30 h, and entered the cell death phase at 30 h. The pH of the culture medium of the three strains gradually decreased with bacterial growth and stabilized after 24 h (Figure 1D). At 36 h, the pH values of the culture medium of strains MC006, MC013, and MC018 were 4.41, 4.64, and 3.79, respectively.

As shown in Figure 1E, the bacterial culture solution and supernatant of the three *Lactobacillus* strains exhibited antibacterial activity against the four *E. coli* test strains. Notably, as compared to the other two *Lactobacillus* strains, *L. fermentum* MC018 exhibited higher antibacterial activity against *E. coli* XH197291, *E. coli* QE191291, *E. coli* BA220820, and *E. coli* BA220826, with mean inhibitory zone diameters of 23.17 ± 0.76 mm, 20.60 ± 0.25 mm, 22.17 ± 0.29 mm, and 22.00 ± 0.68 mm, respectively (Table 1).

**Table 1:** The inhibitory effects of the isolated *Lactobacillus* strains against four *E. coli* strains.

*E. coli* strains	Diameter of inhibition zone (mm)					
	Suspension			Supernatant		
	MC006	MC013	MC018	MC006	MC013	MC018
XH197291	18.33 ± 0.29^c^	18.50 ± 0.50^c^	23.17 ± 0.76^a^	17.50 ± 0.50^d^	19.63 ± 0.32^b^	20.17 ± 0.29^b^
QE191291	16.50 ± 0.32^b^	16.00 ± 0.50^b^	20.60 ± 0.25^a^	20.50 ± 0.50^a^	19.50 ± 0.50^a^	20.27 ± 0.30^a^
BA220820	12.17 ± 0.29^e^	17.00 ± 0.50^d^	22.17 ± 0.29^a^	17.83 ± 0.30^c^	18.17 ± 0.29^c^	21.00 ± 0.50^b^
BA220826	19.67 ± 0.29^b^	16.00 ± 0.32^d^	22.00 ± 0.68^a^	18.97 ± 0.45^b^	18.10 ± 0.17^c^	21.63 ± 0.65^a^

Data within the same row with different superscripts differ significantly (*p* < 0.05).

The survival rates of strains MC006, MC013, and MC018 under pH 2.0 acidic conditions were 39.2% ± 2.3, 32.8% ± 1.8, and 73.8% ± 1.6%, respectively, and their survival rates under 0.3% bile salt conditions were 43.2% ± 4.5, 33.6% ± 3.1, and 75.0% ± 2.1%, respectively (Figure 1F). Additionally, the hydrophobicity level (Table 2) of strains MC006, MC013, and MC018 were 15.27% ± 1.65, 14.89% ± 1.47, and 75.02% ± 3.20%, respectively, and strain MC018 showed the highest hydrophobicity level. The auto-aggregation levels (Table 2) of strains MC006, MC013, and MC018 were 17.26% ± 1.33, 41.71% ± 1.97, and 90.77% ± 0.59%, respectively, with strain MC018 showing the maximum aggregation percentage.

**Table 2:** Comparison of hydrophobicity and auto-aggregation aggregation.

*Lactobacillus* strains	Hydrophobicity (%)	Auto-aggregation (%)
MC006	15.27 ± 1.65^b^	17.26 ± 1.33^c^
MC013	14.89 ± 1.47^b^	41.71 ± 1.97^b^
MC018	75.02 ± 3.20^a^	90.77 ± 0.59^a^

Data within the same column with different superscripts differ significantly (*p* < 0.05).

*In vitro* biosafety of strains MC006, MC013 and MC018 was confirmed by a negative result for blood hemolytic activity (Supplementary Figure S1). Additionally, none of the geese treated with strains MC006, MC013, and MC018 exhibited clinical signs of illness.

### 3.3 Establishment of a goose model of *E. coli* infection

**Figure 2:**
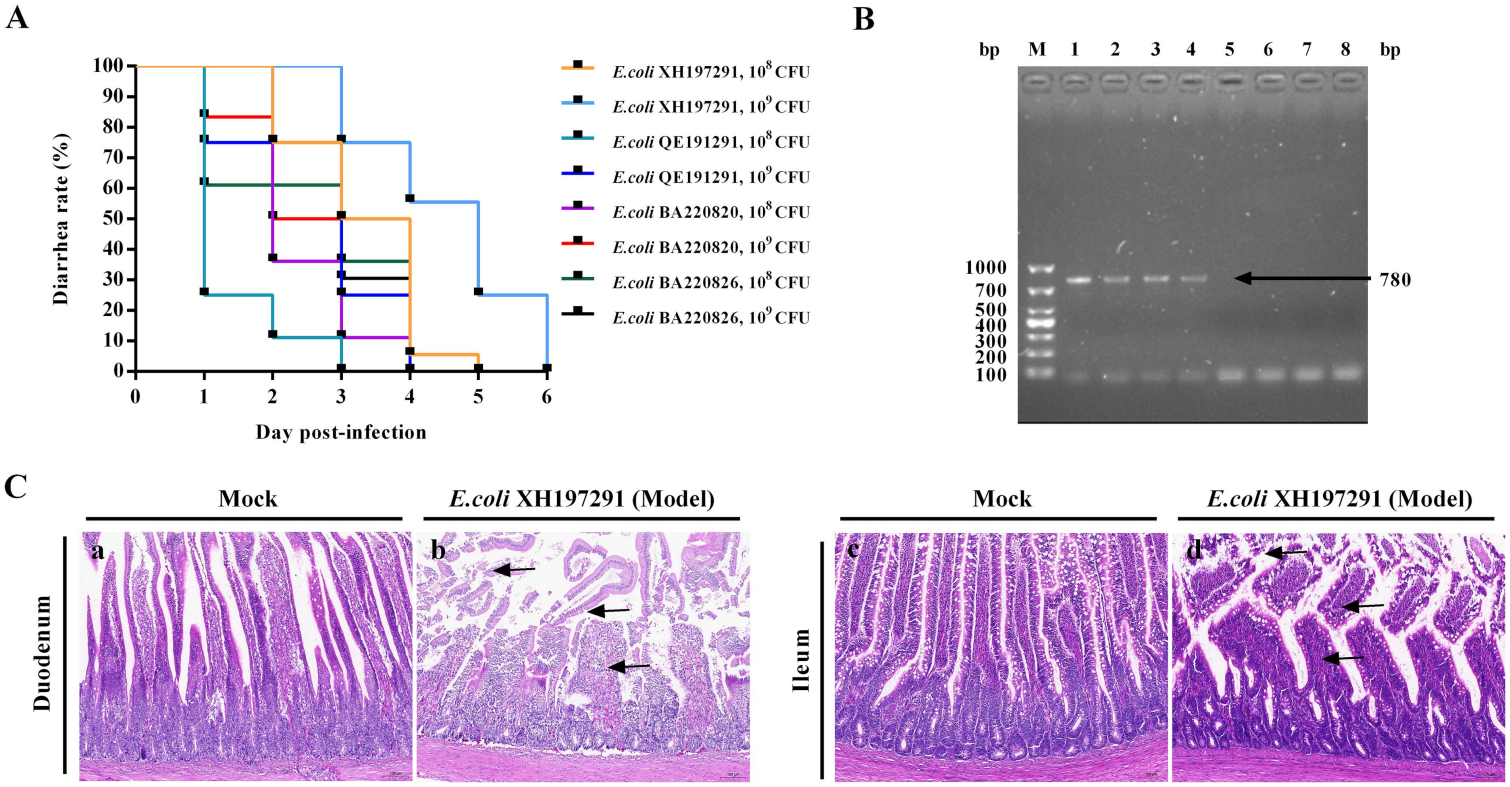
Establishment of a goose model of *E. coli* infection. **(A)** Diarrhea rate; **(B)** PCR detection of *Stx2f* gene. Lane M: 1000 bp DNA marker, line 1: *E. coli* XH197291 was used as the positive control, line 2–4: the fecal swab samples of the geese in the model group, line 5–7: the fecal swab samples of the geese in the mock-infected group, line 8: sterile distilled water was used as the negative control; **(C)** Histological lesions of the duodenum and ileum of the geese in the mock-infected group (a, c) and the model group (b, d).

Geese were infected with *E. coli* XH197291, *E. coli* QE191291, *E. coli* BA220820, and *E. coli* BA220826 by administration through oral gavage. At 24 hpi, geese in each infection group had different degrees of diarrhea. Notably, the diarrhea rate of the *E. coli* XH197291 group (1 mL, 1 × 10^9^ CFU/mL) was 100% within 48 hpi (Figure 2A). Compared to the other infection groups, the duration of diarrhea in geese from the *E. coli* XH197291 group was the longest (5 d) (Figure 2A). As shown in Figure 2B, the *Stx2f* gene was detected in the anal swab samples of geese from the *E. coli* XH197291-infected group, but not in geese from the mock-infected group.

The duodenums of mock-infected geese showed no significant histological lesions (Figures 2C–a,c). *E. coli* XH197291-infected geese showed necrosis and shedding of mucosal epithelial cells, breakage of intestinal villi, and inflammatory cell infiltration (Figures 2C–b). Moreover, the ileums of *E. coli* XH197291-infected geese showed degeneration and atrophy of intestinal villi, necrosis and shedding of mucosal epithelial cells, and inflammatory cell infiltration (Figures 2C–d).

### 3.4 *L. fermentum* MC018 reduced the rate and duration of diarrhea in geese infected with *E. coli* XH197291

**Figure 3:**
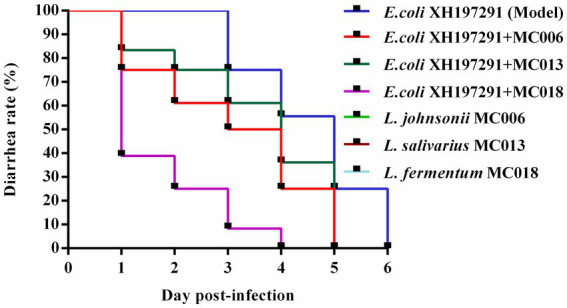
Effect of oral supplementation of the isolated *Lactobacillus* strains on the diarrhea rate of geese infected with *E. coli*.

All three *Lactobacillus* strains reduced the diarrhea rate of *E. coli* XH197291-infected geese (Figure 3). Compared to geese in the other *Lactobacillus* treatment groups and the model group, geese in the *L. fermentum* MC018 group had the lowest diarrhea rate at 24, 48, and 72 hpi (39, 25, and 8%, respectively). Additionally, compared to the model group, the *L. fermentum* MC018 group showed a reduction in the duration of diarrhea to 3 days. None of the geese treated with strains MC006, MC013, and MC018 showed diarrhea.

### 3.5 *L. fermentum* MC018 reduced *E. coli* count and increased LAB count in *E. coli*-infected geese

**Figure 4:**
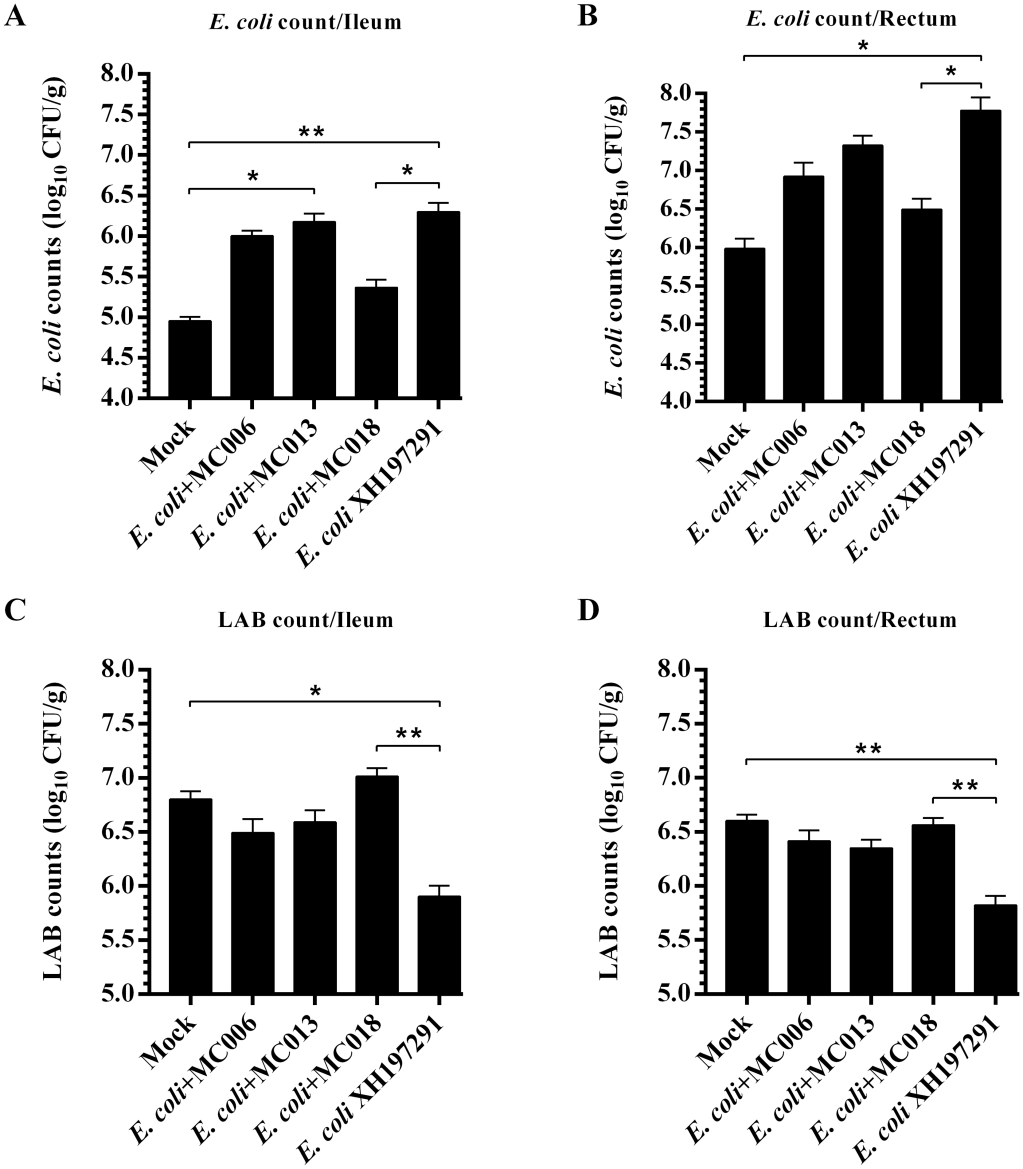
Effects of *L. fermentum* MC018 on the counts of *E. coli* and lactic acid bacteria (LAB) in the ileum and rectum of geese infected with *E. coli* XH197291. **(A)** *E. coli* count in the ileum; **(B)** *E. coli* count in the rectum; **(C)** LAB count in the ileum; **(D)** LAB count in the rectum; *p < 0.05, **p < 0.01 compared with the mock-infected group. Data are presented as mean ± SD (n = 6 per group) and analyzed using one-way ANOVA.

Compared to the mock-infected group, the model group exhibited significantly increased *E. coli* counts in both ileal and rectal contents, concomitant with a marked reduction in LAB populations (Figure 4). Notably, *L. fermentum* MC018 treatment significantly reduced *E. coli* count (Figures 4A,B) and increased LAB count (Figures 4C,D) in *E. coli*-infected geese; however, the other two *Lactobacillus* strains did not significantly affect *E. coli* and LAB counts.

### 3.6 *L. fermentum* MC018 ameliorated *E. coli* XH197291-induced histological lesions in the duodenum and ileum

**Figure 5:**
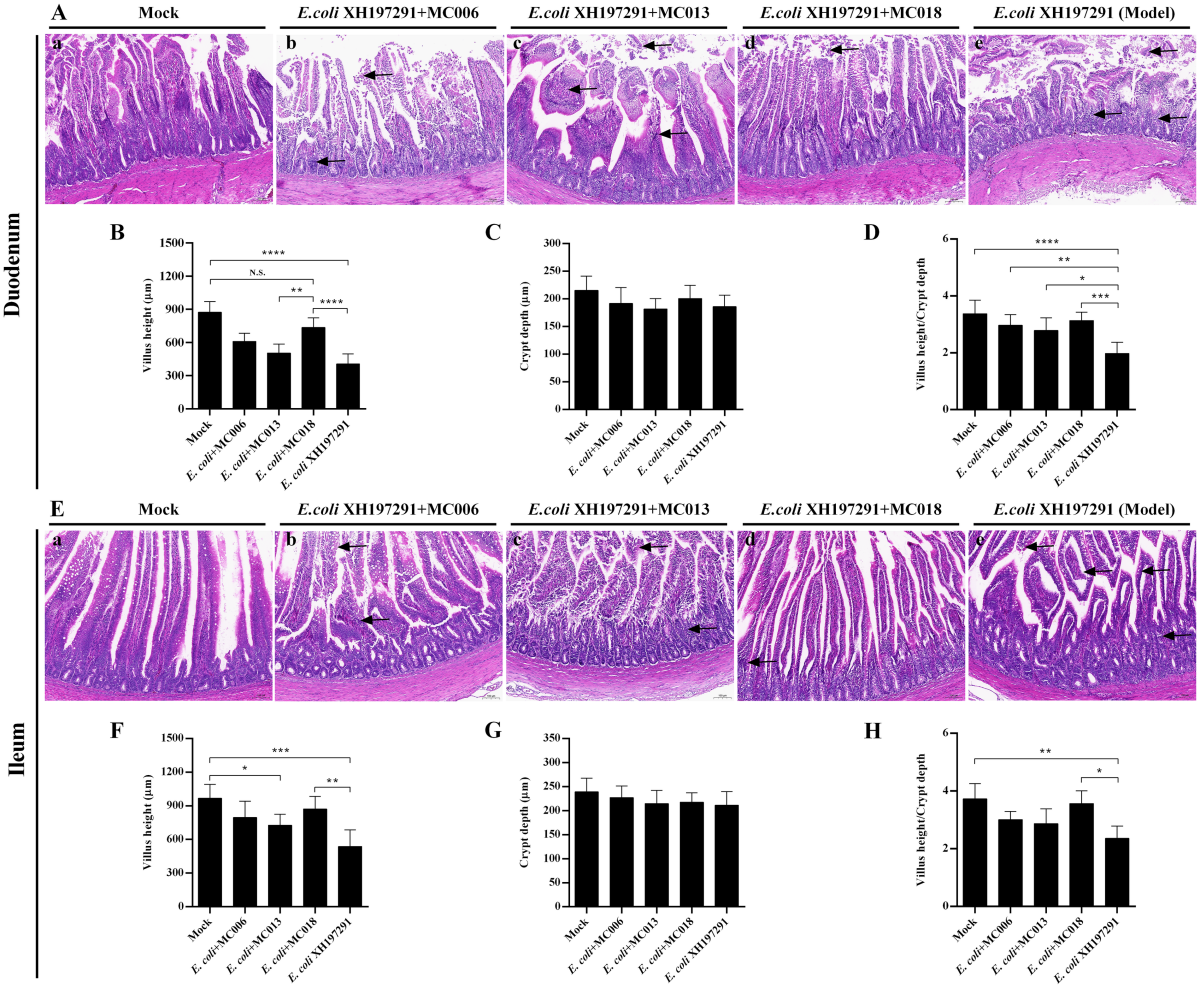
Effect of oral supplementation of the isolated *Lactobacillus* strains on intestinal histopathological changes of geese with *E. coli* infection. **(A)** Histological lesions in the duodenum; **(B)** Villus heights of the duodenum; **(C)** Crypt depths of the duodenum; **(D)** The ratio of villus height/crypt depth (V/C) in the duodenum; **(E)** Histological lesions in the ileum; **(F)** Villus heights of the ileum; **(G)** Crypt depths of the ileum; **(H)** The V/C ratio in the ileum; (a) The mock-infected group, (b) *L. johnsonii* MC006 group, (c) *L. salivarius* MC013 group, (d) *L. fermentum* MC018 group, (e) The model group (*E. coli* XH197291); N. S.: not significant, *p < 0.05, **p < 0.01, ***p < 0.001, ****p < 0.0001 compared with the mock-infected group. Data are presented as mean ± SD (n = 6 per group) and analyzed using one-way ANOVA.

Compared to the *E. coli XH197291*-infected group, the geese groups with *L. johnsonii* MC006, *L. salivarius* MC013, or *L. fermentum* MC018 oral supplementation displayed amelioration of histological lesions in both duodenum and ileum (Figure 5). The mock-infected group showed no significant histological lesions (Figures 5A–a,E–a). *E. coli* XH197291 infection also significantly decreased the villus heights of the duodenum (*p* < 0.0001, Figure 5B) and ileum (*p* < 0.001, Figure 5F). Notably, compared to the *E. coli* XH197291-infected group, the *L. fermentum* MC018 group showed a significant increase in the villus heights of the duodenum (*p* < 0.0001, Figure 5B) and ileum (*p* < 0.01, Figure 5F). However, no significant differences were noted in the crypt depths of the duodenum and ileum between the groups. Moreover, oral supplementation of *L. johnsonii* MC006, *L. salivarius* MC013, or *L. fermentum* MC018 significantly increased the V/C ratio in the duodenum as compared to that in the *E. coli*-infected group (Figure 5D). However, oral supplementation of *L. fermentum* MC018 alone significantly increased the V/C ratio in the ileum (*p* < 0.05, Figure 5H).

### 3.7 *L. fermentum* MC018 improved the intestinal barrier in *E. coli* XH197291-infected geese

**Figure 6:**
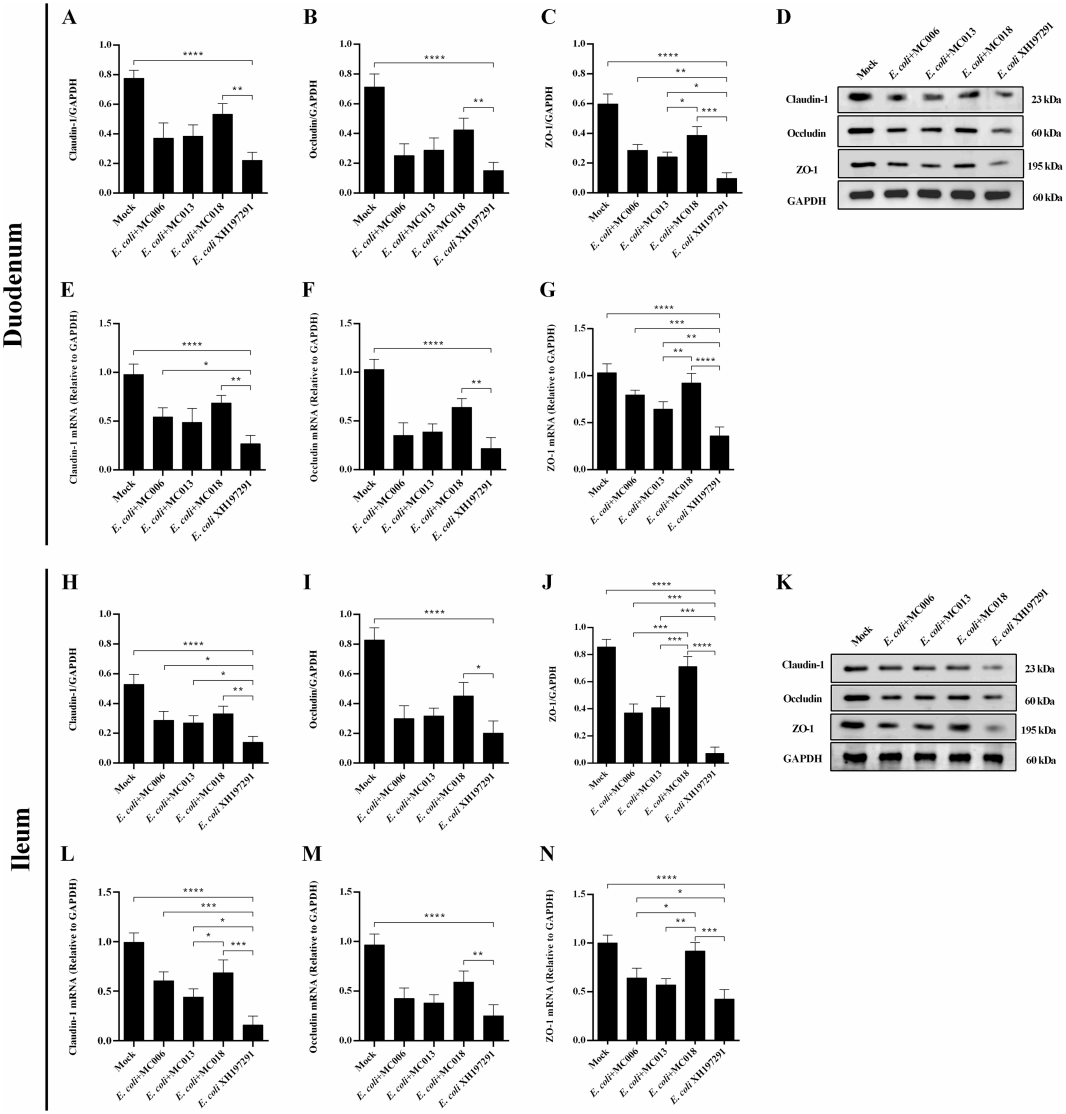
Effect of oral supplementation of the isolated *Lactobacillus* strains on the levels of Claudin-1, Occludin, and ZO-1 in the duodenum and ileum of geese with *E. coli* infection. The protein levels of Claudin-1 **(A)**, Occludin **(B)**, and ZO-1 **(C)** in the duodenum; The mRNA levels of Claudin-1 **(E)**, Occludin **(F)**, and ZO-1 **(G)** in the duodenum; The protein levels of Claudin-1 **(H)**, Occludin **(I)**, and ZO-1 **(J)** in the ileum; The mRNA levels of Claudin-1 **(L)**, Occludin **(M)**, and ZO-1 **(N)** in the ileum; The representative results of Western blot analysis of Claudin-1, Occludin, and ZO-1 in the duodenum **(D)** and ileum **(K)**; *p < 0.05, **p < 0.01, ***p < 0.001, ****p < 0.0001 compared with the mock-infected group. Data are presented as mean ± SD (*n* = 6 per group) and analyzed using one-way ANOVA.

**Figure 7:**
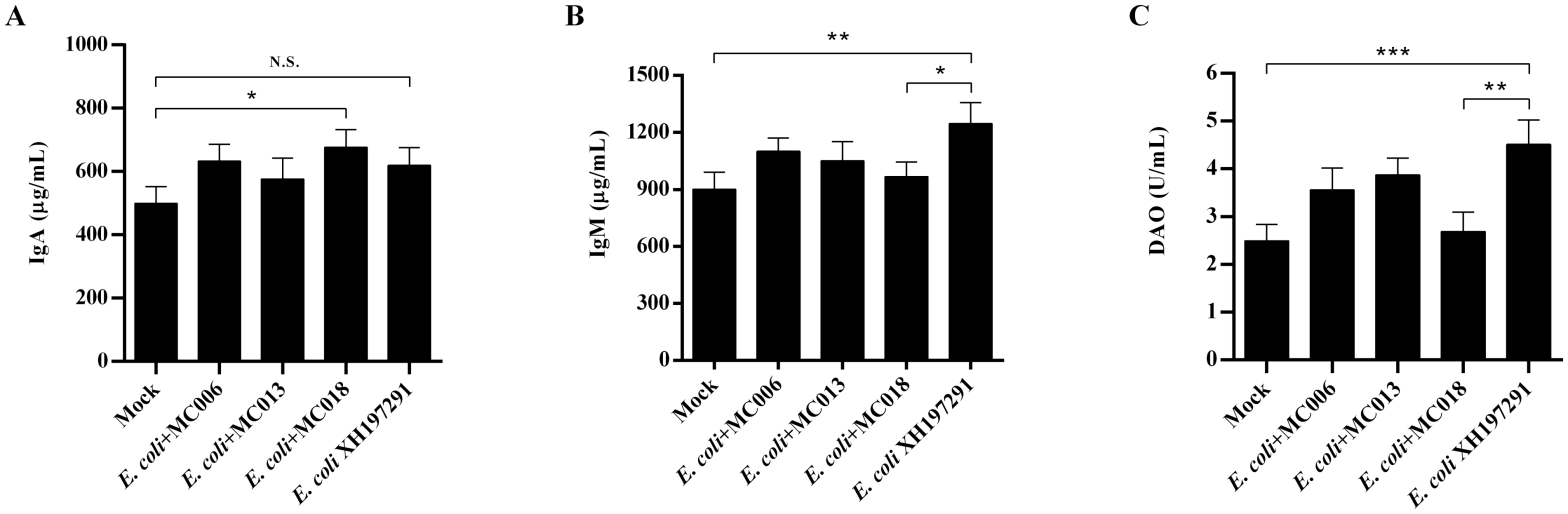
Effect of oral supplementation of the isolated *Lactobacillus* strains on the levels of IgA, IgM, and DAO in the serum of geese with *E. coli* infection. **(A)** The serum IgA level; **(B)** The serum IgM level; **(C)** The serum DAO level. N.S.: not significant, *p < 0.05, **p < 0.01, ***p < 0.001 compared with the mock-infected group. Data are presented as mean ± SD (*n* = 6 per group) and analyzed using one-way ANOVA.

The results of Western blot analysis showed that the protein levels of Claudin-1, Occludin, and ZO-1 in the duodenum and ileum were significantly decreased in the *E. coli* XH197291-infected group as compared to those in the mock-infected group (Figure 6). Notably, *L. fermentum* MC018 significantly restored the protein levels of Claudin-1, Occludin, and ZO-1 in both duodenum (Figures 6A–C) and ileum (Figures 6H–J) as compared to those in the *E. coli*-infected group. Compared to *L. salivarius* MC013, *L. fermentum* MC018 significantly increased the expression levels of ZO-1 in the duodenum (*p* < 0.05, Figure 6C) and ileum (*p* < 0.001, Figure 6J). Additionally, ZO-1 expression was significantly upregulated in the ileum of the *L. fermentum* MC018 group as compared to that in the *L. johnsonii* MC006 group (*p* < 0.001, Figure 6J). The qRT-PCR data also confirmed these findings, showing that *L. fermentum* MC018 supplementation notably decreased the mRNA levels of Claudin-1, Occludin, and ZO-1 in both duodenum (Figures 6E–G) and ileum (Figures 6L–N) as compared to those in the *E. coli*-infected group. Serum DAO levels were measured using ELISA kits. *E. coli* infection significantly increased the serum DAO level as compared to that in the mock-infected group (*p* < 0.001, Figure 7C). In contrast, *L. fermentum* MC018 significantly decreased the serum DAO levels as compared to that in the *E. coli*-infected group (*p* < 0.01, Figure 7C).

### 3.8 *L. fermentum* MC018 decreased the levels of inflammatory cytokines in the duodenum and ileum of geese infected with *E. coli* XH197291

**Figure 8:**
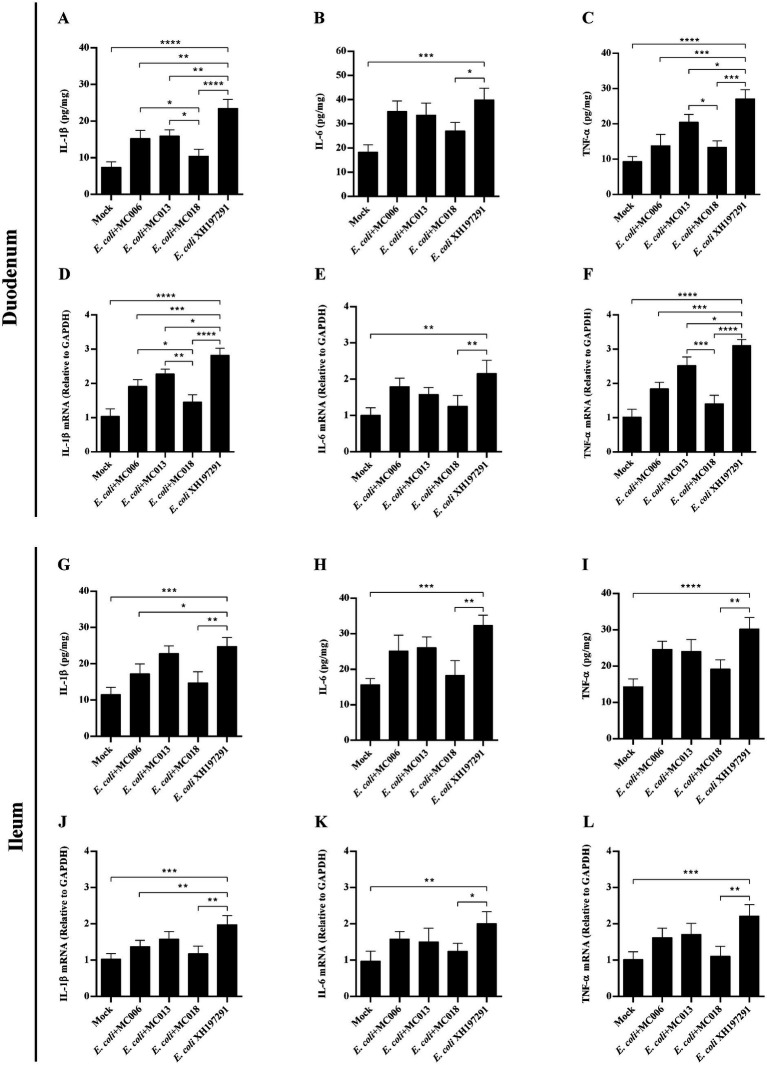
Effect of oral supplementation of the isolated *Lactobacillus* strains on the levels of IL-1β, IL-6, and TNF-*α* in the duodenum and ileum of geese with *E. coli* infection. The protein levels of IL-1β **(A)**, IL-6 **(B)**, and TNF-α **(C)** in the duodenum; The mRNA levels of IL-1β **(D)**, IL-6 **(E)**, and TNF-α **(F)** in the duodenum; The protein levels of IL-1β **(G)**, IL-6 **(H)**, and TNF-α **(I)** in the ileum; The mRNA levels of IL-1β **(J)**, IL-6 **(K)**, and TNF-α **(L)** in the ileum; *p < 0.05, **p < 0.01, ***p < 0.001, ****p < 0.0001 compared with the mock-infected group. Data are presented as mean ± SD (*n* = 6 per group) and analyzed using one-way ANOVA.

ELISA analysis showed that the protein levels of IL-1β, IL-6, and TNF-*α* in both duodenum and ileum were significantly elevated in the *E. coli* XH197291-infected group as compared to those in the mock-infected group (Figure 8). More importantly, *L. fermentum* MC018 supplementation remarkably reduced the protein levels of IL-1β, IL-6, and TNF-α in both duodenum and ileum as compared to those in the *E. coli*-infected group. Additionally, compared to *L. salivarius* MC013, *L. fermentum* MC018 significantly decreased the expression of IL-1β (*p* < 0.05, Figure 8A) and TNF-α (*p* < 0.05, Figure 8C) in the duodenum. The IL-1β expression level was significantly decreased in the duodenum of the *L. fermentum* MC018 group when compared with that in the duodenum of the *L. johnsonii* MC006 group (*p* < 0.05, Figure 8A). The qRT-PCR data also confirmed these findings, showing that *L. fermentum* MC018 supplementation notably decreased the mRNA levels of IL-1β, IL-6, and TNF-α in both duodenum (Figures 8D–F) and ileum (Figures 8J–L) as compared to those in the *E. coli*-infected group.

### 3.9 Serum IgA and IgM levels

ELISA analysis showed that *E. coli* XH197291 infection significantly increased the serum IgM levels (*p* < 0.01) but did not significantly affect the serum IgA levels compared to mock infection (Figures 7A,B). Notably, the serum IgM levels were remarkably decreased in the *L. fermentum* MC018 supplementation group as compared to those in the *E. coli*-infected group (*p* < 0.05, Figure 7B). Moreover, IgA levels were significantly increased in the *L. fermentum* MC018 group when compared with those in the mock-infected group (*p* < 0.05, Figure 7A).

### 3.10 *L. fermentum* MC018 increased the ADG and reduced the FCR in *E. coli* XH197291-infected geese during 8–21 d of age

The *L. salivarius* MC013 and *L. fermentum* MC018 groups showed significant differences in the FCR during 1 to 7 d (*p* < 0.05, Table 3). *E. coli* XH197291 infection significantly reduced the ADG (*p* < 0.05) and increased the FCR (*p* < 0.05) compared to mock infection during 8 to 21 d. More importantly, *L. fermentum* MC018 significantly increased the ADG and reduced the FCR compared to the *E. coli*-infected group (Table 3).

**Table 3:** Effect of oral supplementation of the isolated *Lactobacillus* strains on growth performance of geese infected with *E. coli*.

Groups	1–7 d			8–21 d		
	ADG (g)	ADFI (g)	FCR	ADG (g)	ADFI (g)	FCR
Mock	28.05 ± 3.30	34.50 ± 6.10	1.23 ± 0.12^ab^	51.51 ± 6.41^b^	73.14 ± 10.08	1.42 ± 0.10^a^
MC006	27.93 ± 2.87	31.58 ± 3.56	1.13 ± 0.11^ab^	45.40 ± 6.15^ab^	73.54 ± 10.95	1.62 ± 0.14^ab^
MC013	25.56 ± 2.84	33.80 ± 4.50	1.32 ± 0.12^b^	43.89 ± 3.44^ab^	74.17 ± 6.81	1.69 ± 0.15^ab^
MC018	31.10 ± 2.97	30.93 ± 5.16	0.99 ± 0.10^a^	49.52 ± 5.45^b^	71.81 ± 8.90	1.46 ± 0.16^a^
*E. coli*	25.98 ± 1.80	31.41 ± 3.04	1.21 ± 0.15^ab^	37.78 ± 4.62^a^	66.49 ± 9.13	1.77 ± 0.22^b^

Data within the same column with different superscripts differ significantly (*p* < 0.05). ADG, average daily gain; ADFI, average daily feed intake; FCR, feed conversion ratio.

## 4 Discussion

One of the promising strategies to prevent and treat APEC infection is diet supplementation with *Lactobacillus* probiotics (3, 17). To exert its health-improving effects, *Lactobacillus* must overcome obstacles such as the presence of acid in the stomach and bile in the intestine. In the present study, *L. johnsonii* MC006, *L. salivarius* MC013, and *L. fermentum* MC018 exhibited resistance to degradation by acid and bile, suggesting that these strains could possibly survive under acidic conditions in the stomach and intestine. Additionally, the ability of bacteria to auto-aggregate reflects their persistence in the gut, and this aggregation eliminates or reduces pathogen adherence (22). Surface hydrophobicity represents the capacity of *Lactobacillus* to adhere to and colonize the intestinal tract, with a higher hydrophobicity level indicating stronger adhesion ability. A previous study reported that the auto-aggregation level of *L. acidophilus* LA7 and *L. plantarum* Lp9 was 46.5 and 31%, respectively, which confirmed their ability to auto-aggregate (27). *Lactobacillus* with surface hydrophobicity of >50% is strongly hydrophobic (22). In the present study, we found that the auto-aggregation and hydrophobicity levels of *L. fermentum* MC018 were 90.77 and 75.02%, respectively; this finding indicated the ability of this strain to survive and colonize the gastrointestinal tract, thus confirming its probiotic properties.

To investigate the protective effects of goose-derived *Lactobacillus* against *E. coli* infection in geese, suitable animal models are essential and may serve as useful experimental tools. However, to date, few studies have established a goose model of *E. coli* infection. As reported previously, both intraperitoneal injection and oral administration can successfully induce *E. coli* infection in broilers (3, 24, 28). The infected broilers exhibited typical symptoms, including loss of appetite and diarrhea, and some broilers died due to infection. *E. coli* infection caused intestinal damage and inflammation and decreased the growth performance of broilers (28). In the present study, Zi geese were infected with *E. coli* XH197291, *E. coli* QE191291, *E. coli* BA220820, or *E. coli* BA220826 through oral gavage. Consistent with the results of previous studies, we found that *E. coli* XH197291-infected geese exhibited depression, loss of appetite, diarrhea, intestinal damage, reduced ADG, and increased FCR. More importantly, compared to the other infection groups, the *E. coli* XH197291 infection group showed a diarrhea rate of 100% within 48 hpi, with the longest time of diarrhea duration (up to 5 d). These findings confirmed the successful establishment of a Zi goose model of *E. coli* XH197291 infection and provided a basis to evaluate the protective effects of *Lactobacillus* supplementation against *E. coli* infection.

*Lactobacillus* supplementation in *E. coli*-infected poultry can reduce diarrhea rate and mortality, alleviate intestinal injury, ameliorate intestinal barrier function, and improve growth performance. According to previous studies, *L. acidophilus* supplementation reduced the mortality of *E. coli*-infected broilers and elevated serum DAO levels and mRNA expression levels of Occludin and ZO-1 in the jejunum and ileum (3). Compound feed additive containing *L. acidophilus* reduced the diarrhea rate in *E. coli*-infected broilers (24). Oral administration of *L. acidophilus* and *Bacillus subtilis* alleviated the pathological damage in the intestine of *E. coli-*infected broilers without reducing the diarrhea rate (29). In the present study, we found that *L. fermentum* MC018 supplementation not only alleviated intestinal injury of geese with *E. coli* infection and elevated the levels of Claudin-1, Occludin, ZO-1, and DAO, but also reduced the diarrhea rate and *E. coli* count and increased LAB count in both ileum and rectum. These results suggest that *L. fermentum* MC018 could be a candidate probiotic to prevent and alleviate diarrhea and intestinal damage and improve intestinal barrier function in *E. coli*-infected geese. The villus height and V/C ratio are the relevant measures of intestinal absorptive capacity and intestinal health (3). A decrease in the villus height and V/C ratio diminishes the nutrient absorption capacity of the small intestine, reduces disease resistance, and lowers the growth performance of poultry (17, 28). Similarly, in the present study, *E. coli* XH197291 infection decreased the villus height and V/C ratio in the duodenum and ileum of geese; moreover, this reduction was accompanied by decreased ADG and increased FCR of infected geese. More importantly, compared to the *E. coli*-infected group, *L. fermentum* MC018 significantly enhanced the V/C ratios in the duodenum and ileum and decreased FCR; this result was consistent with the findings of previous studies that reported an improvement in the V/C ratio and growth performance of broilers treated with *L. acidophilus* (CGMCC 14437) or *L. plantarum* B1 (3, 17).

IL-1β and TNF-*α* are two important proinflammatory cytokines that participate in the regulation of inflammatory response at the early infection period (30). *E. coli* infection increased IL-1β and TNF-α levels in the jejunum of broilers. (3, 31). Our results also showed that *E. coli* XH197291 infection elevated IL-1β and TNF-α levels in the duodenum and ileum of Zi geese. Notably, *L. fermentum* MC018 decreased IL-1β and TNF-α levels in the duodenum and ileum of *E. coli-*infected geese; this finding was consistent with the conclusions of previous studies that reported a reduction in IL-1β and TNF-α levels in broilers treated with *L. acidophilus* or *L. plantarum* (3, 17). These findings suggest that *L. fermentum* MC018 exerts an anti-inflammatory effect on the gut of *E. coli*-infected geese. IL-6 shows a critical immunomodulatory effect on B cell activation, antibody secretion, and the activation and proliferation of cytotoxic T cells (32). A previous study reported that *L. acidophilus* supplementation did not significantly affect *IL-6* gene expression in the jejunum and spleen of *E. coli*-infected broilers (3). In contrast, the present study found that oral supplementation of *L. fermentum* MC018 substantially downregulated IL-6 mRNA expression in the duodenum and ileum of infected geese as compared to that in the *E. coli*-infected group. This might be due to differences in animal models, *Lactobacillus* species, and host status.

The immune globulins (Ig) such as IgA and IgM are produced by mature B cells in response to antigenic stimulation and play a pivotal role in various immune responses (33, 34). The present study found that *E. coli* challenge increased serum IgA and IgM levels at the early stage of infection; this finding is consistent with the results of Wu et al. (3). When geese are in the early stages of infection, *E. coli* acts as a foreign antigen that stimulates B cells to regulate the immune system and produce antibodies (3). In addition, *L. acidophilus* supplementation did not significantly affect the serum levels of IgA and IgM in *E. coli*-infected broilers. However, our findings revealed that *L. fermentum* MC018 remarkably decreased the serum IgM levels as compared to that in the *E. coli*-infected group. IgM can regulate immunity, sterilize, agglutinate, and activate the complement system in the early stages of pathogen infection (35). The decreased IgM levels observed in this study might be due to a decrease in the number of pathogens in the intestines of *E. coli*-infected geese fed *L. fermentum* MC018 during the infection period, which was insufficient to stimulate the immune system to produce more specific Ig.

The combined use of multiple probiotic strains demonstrates favorable synergistic effects. In mice, the co-administration of *L. acidophilus* NCFM and *L. plantarum* Lp-115 effectively limited *Helicobacter pylori* colonization on gastric mucosa while also suppressing inflammation (36). Dietary supplementation with *L. acidophilus* and *B. subtilis* enhanced intestinal barrier function and preserved immunological homeostasis in hens (9). Additionally, *L. johnsonii* relieved enterohaemorrhagic *E. coli*-induced diarrhea and altered the structure of intestinal flora in rats (37). In broilers challenged with coccidia and *Clostridium perfringens*, dietary *L. fermentum* improved intestinal health by strengthening the intestinal barrier and reducing inflammation (38). Similarly, our research discovered that oral supplementation with *L. johnsonii* MC006 or *L. fermentum* MC018 ameliorated diarrhea and intestinal histological lesions in *E. coli*-infected geese, as well as reduce the expression of pro-inflammatory cytokines (IL-1β and TNF-*α*) in the duodenum and ileum. However, the potential synergistic interaction between *L. johnsonii* MC006 and *L. fermentum* MC018 needs to be further studied and confirmed.

## 5 Conclusion

Based on the *in vitro* study, goose-derived *L. fermentum* MC018 appears to be a promising probiotic because of its ability to resist acid and bile degradation, exert antibacterial activity on *E. coli*, and adhere strongly to the intestinal tract. Additionally, the *in vivo* study revealed that oral supplementation of *E. coli*-infected geese with *L. fermentum* MC018 alleviated diarrhea, intestinal damage, and inflammation; reduced *E. coli* counts in both ileum and rectum; increased intestinal LAB population, and improved intestinal barrier function and growth performance. These findings provide a scientific basis to explore *Lactobacillus* supplementation-based strategies to prevent *E. coli* infection and promote growth in geese.

## Data Availability

The original contributions presented in the study are included in the article/Supplementary material, further inquiries can be directed to the corresponding author.
