# Supplementary figures and images for: Oral supplementation with Lactobacillus fermentum MC018 improves intestinal health, immune response, and growth performance of Zi geese infected with Escherichia coli XH197291
Source: Front Vet Sci. 2025 Sep 2;12:1666985. doi: 10.3389/fvets.2025.1666985 (PMC12436130; doi:10.3389/fvets.2025.1666985)

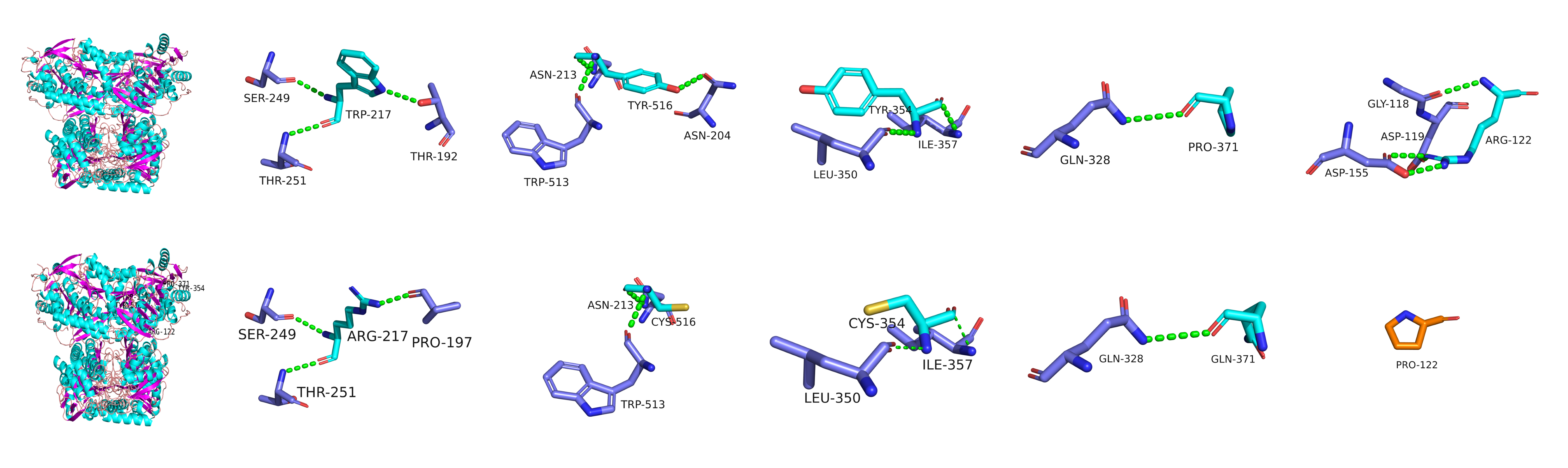

Supplement: Supplementary file 1 [file Image_1.png]
