# Supplementary material for: Oral supplementation with Lactobacillus fermentum MC018 improves intestinal health, immune response, and growth performance of Zi geese infected with Escherichia coli XH197291
Source: Front Vet Sci. 2025 Sep 2;12:1666985. doi: 10.3389/fvets.2025.1666985 (PMC12436130; doi:10.3389/fvets.2025.1666985)
Supplement: Supplementary file 2 [file Table_1.docx]

**Supplementary Table S1** Nutrient levels of the commercial gosling diets

| Nutrient levels | Contents (%) |
| --- | --- |
| Crude protein | 18 |
| Crude fibre | 7 |
| Crude ash | 7 |
| Calcium | 1 |
| Phosphorus | 0.45 |
| NaCl | 0.35 |
| Methionine | 0.35 |
