# Supplementary material for: Oral supplementation with Lactobacillus fermentum MC018 improves intestinal health, immune response, and growth performance of Zi geese infected with Escherichia coli XH197291
Source: Front Vet Sci. 2025 Sep 2;12:1666985. doi: 10.3389/fvets.2025.1666985 (PMC12436130; doi:10.3389/fvets.2025.1666985)
Supplement: Supplementary file 3 [file Table_2.docx]

**Supplementary** **Table S2** Primer sequences used for qRT-PCR

| Gene | Forward primer | Reverse primer |
| --- | --- | --- |
| Claudin1 | CATTGTGGAGTGGCCTTGGAGTG | AGCTGGTTGGGTGAACTGAATGC |
| Occludin | CCACCGCCACCATGTTCAGC | GCCGTAGTCGTAGCCGTAATCAC |
| ZO-1 | TTTCCCCGTCGCCTGAATCAAAC | GCCCGCTTGTGGTTGGTAAGAG |
| IL-1β | CAGAAGAAGCCTCGCCTGGATTC | GCCTCCGCAGCAGTTTGGTC |
| IL-6 | AAGCATCTGGCAACGACGATAAGG | TGTGAGGAGGGATTTCTGGGTAGC |
| TNF-α | CGCACCAGCCACGGACATTC | GGTGAGGTAGGCAGAGGTCAGAG |
| GAPDH | GGTAGTGAAGGCTGCTGCTGATG | GGAGGAATGGCTGTCACCGTTG |
